# Cardiovascular outcomes with semaglutide by severity of chronic kidney disease in type 2 diabetes: the FLOW trial

**DOI:** 10.1093/eurheartj/ehae613

**Published:** 2024-08-30

**Authors:** Kenneth W Mahaffey, Katherine R Tuttle, Mustafa Arici, Florian M M Baeres, George Bakris, David M Charytan, David Z I Cherney, Gil Chernin, Ricardo Correa-Rotter, Janusz Gumprecht, Thomas Idorn, Giuseppe Pugliese, Ida Kirstine Bull Rasmussen, Søren Rasmussen, Peter Rossing, Ekaterina Sokareva, Johannes F E Mann, Vlado Perkovic, Richard Pratley

**Affiliations:** Stanford Center for Clinical Research, Department of Medicine, Stanford School of Medicine, 300 Pasteur Drive, Grant S-102, Stanford, Palo Alto, CA 94305, USA; Division of Nephrology, University of Washington School of Medicine, Seattle, WA, USA; Providence Medical Research Center, Providence Inland Northwest Health, Spokane, WA, USA; Department of Nephrology, Hacettepe University Faculty of Medicine, Ankara, Turkey; Novo Nordisk A/S, Søborg, Denmark; Department of Medicine, New York University Grossman School of Medicine, New York, NY, USA; Division of Nephrology, University of Toronto, Toronto, ON, Canada; Kaplan Medical Center, Hebrew University of Jerusalem, Rehovot, Israel; National Institute of Medical Sciences and Nutrition, Salvador Zubirán, Mexico City, Mexico; Department of Clinical and Molecular Medicine, Medical University of Silesia, Katowice, Poland; Novo Nordisk A/S, Søborg, Denmark; Department of Clinical and Molecular Medicine, La Sapienza University, Rome, Italy; Novo Nordisk A/S, Søborg, Denmark; Novo Nordisk A/S, Søborg, Denmark; Steno Diabetes Center Copenhagen, Herlev, Denmark; Department of Clinical Medicine, University of Copenhagen, Copenhagen, Denmark; Novo Nordisk A/S, Søborg, Denmark; KfH Kidney Centre, Munich, Germany; Department of Nephrology, Hypertension and Rheumatology, University Hospital, Friedrich-Alexander University, Erlangen, Germany; Faculty of Medicine and Health, University of New South Wales, Sydney, NSW, Australia; AdventHealth Translational Research Institute, Orlando, FL, USA

**Keywords:** Semaglutide, Cardiovascular outcomes, Type 2 diabetes, Chronic kidney disease

## Abstract

**Background and Aims:**

In the FLOW trial, semaglutide reduced the risks of kidney and cardiovascular (CV) outcomes and death in participants with type 2 diabetes and chronic kidney disease (CKD). These prespecified analyses assessed the effects of semaglutide on CV outcomes and death by CKD severity.

**Methods:**

Participants were randomized to subcutaneous semaglutide 1 mg or placebo weekly. The main outcome was a composite of CV death, non-fatal myocardial infarction (MI), or non-fatal stroke (CV death/MI/stroke) as well as death due to any cause by baseline CKD severity. CKD was categorized by estimated glomerular filtration rate < or ≥60 mL/min/1.73 m^2^, urine albumin-to-creatinine ratio < or ≥300 mg/g, or Kidney Disease Improving Global Outcomes (KDIGO) risk classification.

**Results:**

Three thousand, five hundred and thirty-three participants were randomized with a median follow-up of 3.4 years. Low/moderate KDIGO risk was present in 242 (6.8%), while 878 (24.9%) had high and 2412 (68.3%) had very high KDIGO risk. Semaglutide reduced CV death/MI/stroke by 18% [hazard ratio (HR) 0.82 (95% confidence interval 0.68–0.98); *P* = .03], with consistency across estimated glomerular filtration rate categories, urine albumin-to-creatinine ratio levels, and KDIGO risk classification (all *P*-interaction > .13). Death due to any cause was reduced by 20% [HR 0.80 (0.67–0.95); *P* = .01], with consistency across estimated glomerular filtration rate categories and KDIGO risk class (*P*-interaction .21 and .23, respectively). The *P*-interaction treatment effect for death due to any cause by urine albumin-to-creatinine ratio was .01 [<300 mg/g HR 1.17 (0.83–1.65); ≥300 mg/g HR 0.70 (0.57–0.85)].

**Conclusions:**

Semaglutide significantly reduced the risk of CV death/MI/stroke regardless of baseline CKD severity in participants with type 2 diabetes.

## Structured Graphical Abstract

**Structured Graphical Abstract:**
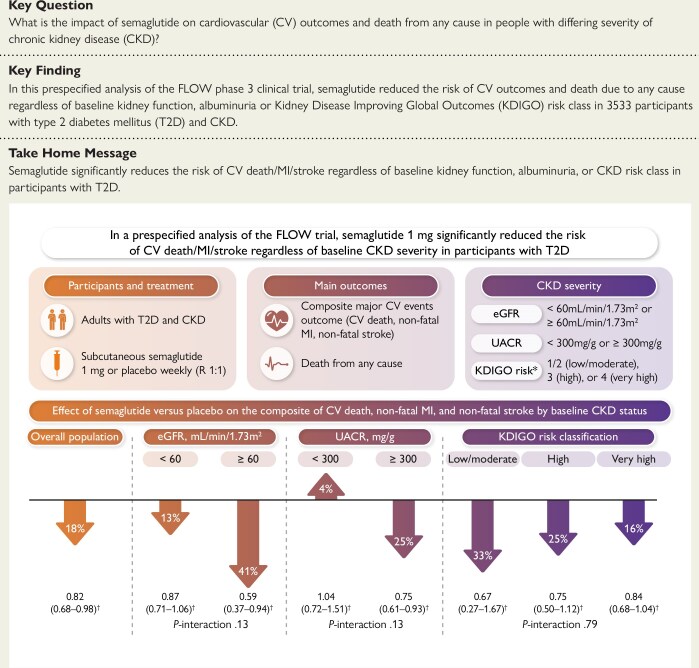
In a prespecified analysis of the FLOW trial, semaglutide 1.0 mg significantly reduced the risk of CV death/MI/stroke regardless of baseline CKD severity in participants with T2D. *KDIGO-defined risk classification: low, moderate, high, and very high risk based on albuminuria and eGFR. ^†^Data are the HR and 95% CI for the composite major CV event outcome. CI, confidence interval; CKD, chronic kidney disease; CV, cardiovascular; eGFR, estimated glomerular filtration rate; HR, hazard ratio; KDIGO, Kidney Disease Improving Global Outcomes; MI, myocardial infarction; R, randomization; T2D, type 2 diabetes; UACR, urine albumin-to-creatinine ratio.

**See the editorial comment for this article ‘Pump, pipes, filter, sugar, weight, and more: the pluripotent prowess of semaglutide’, by S. Verma et al., https://doi.org/10.1093/eurheartj/ehae744.**

## Introduction

People with type 2 diabetes (T2D) and chronic kidney disease (CKD) are at high risk for cardiovascular (CV) events, including CV death, myocardial infarction (MI), and stroke, as well as death due to any cause.^1^ The high risk for CV events and mortality is strongly correlated with the severity of CKD, defined by estimated glomerular filtration rate (eGFR), urine albumin-to-creatinine ratio (UACR), or Kidney Disease Improving Global Outcomes (KDIGO) risk classification using both eGFR and UACR.^2–5^

Renin–angiotensin system inhibitors, sodium–glucose co-transporter 2 inhibitors (SGLT2i), and finerenone provide both kidney protection and reduction of risk of CV events in patients with T2D and high CV risk or CKD.^6–12^ These therapies are an essential part of a foundation of optimal care as recommended by clinical practice guidelines for this population of people with T2D.^13,14^ Despite these evidence-based therapies, there is a clear unmet need for additional effective therapies in this patient population as a high residual risk is still present. Treatment with glucagon-like peptide-1 receptor agonists (GLP-1RAs) has reduced CV risk in several CV outcomes trials.^15–20^ A dedicated kidney outcomes trial, the FLOW (Evaluate Renal Function with Semaglutide Once Weekly) trial, showed that semaglutide reduced the risk of major kidney events by 24%, major CV events by 18%, and death due to any cause by 20%, when compared with placebo, in participants with T2D and CKD.^21^

In the prespecified analyses of the FLOW trial reported here, the composite of CV death, non-fatal MI or non-fatal stroke (hereafter CV death/MI/stroke), and death due to any cause, as well as safety outcomes, were examined by baseline CKD status to better understand if the treatment effects in people with differing severity of CKD are consistent and support similar therapeutic strategies.

## Methods

### Study design

The design and primary results of the FLOW trial have been published previously.^21,22^ The sponsor performed the analyses. The kidney, CV, and death events had been validated by an independent statistical group as part of the validation of the primary analyses previously published.^21^

Adults with T2D (glycated haemoglobin ≤10%) and CKD defined as eGFR 50–75 mL/min/1.73 m^2^ (calculated with serum creatinine using the Chronic Kidney Disease Epidemiology Collaboration 2009 formula)^23^ and UACR >300 to <5000 mg/g or eGFR 25–<50 mL/min/1.73 m^2^ and UACR >100 to <5000 mg/g while receiving a stable maximal labelled or tolerated dose of renin–angiotensin system inhibitors (angiotensin-converting enzyme inhibitor or angiotensin receptor blocker) were eligible to be enrolled. Full inclusion and exclusion criteria have been published previously.^21^

Participants were randomly assigned 1:1 to receive subcutaneous semaglutide 1.0 mg or matching placebo once weekly and followed for CV, kidney, and safety outcomes. The trial was stopped early for efficacy based on a recommendation from the independent Data Monitoring Committee.

### Study outcomes

The main outcome for this prespecified analysis was a composite of CV death/MI/stroke, which was a secondary confirmatory endpoint included in the testing hierarchy. Other outcomes included death due to any cause and safety events. All major CV events and cause of death were adjudicated by an independent committee according to prespecified endpoint definitions and have been published.^21^

The efficacy outcomes for these analyses included those in the prespecified hierarchical testing sequence detailed previously.^21^ All deaths, major adverse CV events, major kidney disease outcomes, and major adverse limb ischaemia events were reviewed by adjudication committees blinded to therapy. The definitions used for the clinical events have been published.^21,22^ Safety was assessed as the number and nature of serious adverse events (SAEs) or those adverse events (AEs) leading to discontinuation of study drug.

### Baseline kidney function

In this analysis, participants were grouped by baseline CKD status according to three methods: (i) eGFR (Chronic Kidney Disease Epidemiology Collaboration 2009) defined as <60 or ≥60 mL/min/1.73 m^2^; (ii) UACR defined as <300 or ≥300 mg/g; and (iii) KDIGO-defined risk classification: low, moderate, high, and very high risk based on albuminuria and eGFR.^2^ Enrolment in the study could be based on historical local laboratory data, but baseline data are from a central laboratory analysis only. UACR was calculated as the geometric mean of two first morning urine voids. No imputations were performed for missing data.

### Statistical analysis

Efficacy analyses were based on the intention-to-treat principle using in-trial data and included all unique randomized participants. Time-to-event outcomes were analysed using a stratified Cox proportional hazards model with randomized treatment (semaglutide or placebo) as a fixed factor and stratified by SGLT2i use at baseline. *P*-values were obtained from a score test in the Cox proportional hazards model. For the primary outcome, the hazard ratio (HR), 95% confidence interval (CI), and *P*-value were adjusted for the group sequential design using the likelihood ratio ordering. Interaction *P*-values were calculated to evaluate the heterogeneity of treatment effect by CKD status. No adjustment was made for multiple comparisons. A two-sided *P*-value of 0.05 was considered significant. The absolute risk difference was estimated using a generalized linear regression model with identity link function on the pseudo-observations from the Aalen–Johansen estimate at Week 156. Treatment and SGLT2i use (yes/no) at baseline were included as fixed factors in the regression model. The estimate of the number needed to treat (NNT) is calculated as 1/(cumulative incidence estimate for placebo minus cumulative incidence estimate for semaglutide 1.0 mg). Absolute risk reductions were reported with 95% CI, and corresponding NNTs were reported if the reduction was in favour of semaglutide as indicated by the 95% CI (not including zero). For the comparison of baseline characteristics, *P*-values were determined using a Kruskal–Wallis test for continuous variables and a *χ*^2^ test for categorical variables. Adverse events were summarized by the proportion of participants with an event using data according to randomly assigned treatment and KDIGO risk class.

All statistical analyses were performed with SAS software, version 9.4 TS1M5 (SAS Institute, Cary, NC, USA).

## Results

In total, 3533 participants were randomly assigned to semaglutide or placebo with a mean follow-up of 3.4 years. Vital status was known in 98.6% of participants, and adherence to randomized treatment was 89%. Baseline CKD status by baseline eGFR, UACR, and KDIGO risk class was known for 3532 participants (99.97%). At baseline, 2813 participants (79.6%) had eGFR <60 mL/min/1.73 m^2^ and 719 participants (20.4%) had eGFR ≥60 mL/min/1.73 m^2^. Additionally, 1113 participants (31.5%) had a baseline UACR <300 mg/g and 2419 participants (68.5%) had a baseline UACR ≥300 mg/g. In terms of KDIGO risk class, 242 participants (6.8%) were low/moderate risk, 878 participants (24.9%) were high risk, and 2412 participants (68.3%) were very high risk.

*Table 1* shows the baseline characteristics of the participants enrolled by KDIGO risk class and randomly assigned treatment. Participants with higher KDIGO risk classes tended to be slightly older, were more likely to be male, and had a slightly lower level of high-density lipoprotein cholesterol.

**Table 1:** Baseline characteristics by KDIGO risk class

Parameter	KDIGO low/moderate risk		KDIGO high risk		KDIGO very high risk	
	Semaglutide 1 mg (*n* = 123)	Placebo (*n* = 119)	Semaglutide 1 mg (*n* = 432)	Placebo (*n* = 446)	Semaglutide 1 mg (*n* = 1211)	Placebo (*n* = 1201)
Age, years	67.0 (62.0–72.0)	66.0 (60.0–72.0)	67.0 (60.0–72.0)	67.0 (62.0–73.0)	68.0 (62.0–73.0)	68.0 (61.0–73.0)
Male sex	78 (63.4)	75 (63.0)	321 (74.3)	312 (70.0)	848 (70.0)	829 (69.0)
Region						
North America	23 (18.7)	34 (28.6)	89 (20.6)	113 (25.3)	310 (25.6)	295 (24.6)
South America	5 (4.1)	8 (6.7)	36 (8.3)	38 (8.5)	80 (6.6)	85 (7.1)
Europe	28 (22.8)	23 (19.3)	134 (31.0)	134 (30.0)	310 (25.6)	334 (27.8)
Africa	8 (6.5)	3 (2.5)	17 (3.9)	8 (1.8)	26 (2.1)	39 (3.2)
Asia	26 (21.1)	28 (23.5)	106 (24.5)	89 (20.0)	346 (28.6)	317 (26.4)
Other	33 (26.8)	23 (19.3)	50 (11.6)	64 (14.3)	139 (11.5)	131 (10.9)
Race						
Asian	28 (22.8)	29 (24.4)	104 (24.1)	84 (18.8)	307 (25.4)	294 (24.5)
Black or African American	3 (2.4)	6 (5.0)	14 (3.2)	18 (4.0)	60 (5.0)	58 (4.8)
White	87 (70.7)	79 (66.4)	290 (67.1)	312 (70.0)	778 (64.2)	777 (64.7)
Other	3 (2.4)	4 (3.4)	17 (3.9)	19 (4.3)	36 (3.0)	46 (3.8)
Not reported	2 (1.6)	1 (0.8)	7 (1.6)	13 (2.9)	30 (2.5)	26 (2.2)
Ethnicity						
Hispanic/Latino	14 (11.4)	21 (17.6)	80 (18.5)	80 (17.9)	179 (14.8)	182 (15.2
Not Hispanic/Latino	103 (83.7)	97 (81.5)	337 (78.0)	346 (77.6)	980 (80.9)	968 (80.6)
Not reported	6 (4.9)	1 (0.8)	15 (3.5)	20 (4.5)	52 (4.3)	51 (4.2)
History of CV events						
No prior MI or stroke	91 (74.0)	90 (75.6)	329 (76.9)	340 (76.7)	924 (77.1)	913 (77.1)
Prior MI or stroke	32 (26.0)	29 (24.4)	99 (23.1)	103 (23.3)	274 (22.9)	271 (22.9)
Tobacco use						
Current smoker	13 (10.6)	13 (10.9)	61 (14.1)	61 (13.7)	149 (12.3)	132 (11.0)
Never smoked	63 (51.2)	67 (56.3)	198 (45.8)	196 (43.9)	621 (51.3)	601 (50.0)
Previous smoker	47 (38.2)	39 (32.8)	173 (40.0)	189 (42.4)	441 (36.4)	468 (39.0)
BMI, kg/m^2^	31.8 (27.5–35.4)	30.1 (27.0–35.0)	31.4 (28.0–35.0)	31.8 (27.6–36.2)	31.2 (27.4–35.4)	31.3 (27.3–35.6)
Body weight, kg	89.0 (77.6–102.0)	85.8 (73.9–97.1)	89.8 (76.0–102.3)	89.1 (75.3–104.0)	87.0 (75.0–101.0)	87.2 (74.3–103.0)
HbA_1c_, %	7.6 (6.9–8.4)	7.6 (6.9–8.5)	7.8 (7.0–8.7)	7.6 (6.9–8.6)	7.6 (6.9–8.5)	7.6 (6.8–8.6)
eGFR, mL/min/1.73 m^2^	66.0 (61.0–73.0)	66.0 (61.0–76.0)	62.0 (52.0–72.0)	61.0 (50.0–68.0)	38.0 (32.0–46.0)	40.0 (33.0–46.0)
UACR, mg/g	137.8 (22.9–240.1)	137.6 (37.2–215.6)	442.9 (171.4–1021.7)	380.2 (171.4–955.1)	751.9 (337.2–1600.0)	723.5 (353.1–1530.2)
hsCRP, mg/L	2.6 (1.2–6.9)	2.5 (1.3–6.8)	2.3 (1.2–5.6)	2.4 (1.1–5.3)	2.5 (1.1–5.6)	2.6 (1.2–6.3)
Total cholesterol, mmol/L	4.5 (3.8–5.3)	4.3 (3.7–5.1)	4.1 (3.5–5.0)	4.2 (3.6–5.0)	4.2 (3.6–5.1)	4.2 (3.5–5.0)
LDL-cholesterol, mmol/L	2.3 (1.7–2.9)	2.2 (1.6–2.7)	2.1 (1.5–2.7)	2.1 (1.6–2.8)	2.2 (1.6–2.8)	2.1 (1.6–2.8)
HDL-cholesterol, mmol/L	1.1 (1.0–1.3)	1.1 (0.9–1.3)	1.1 (0.9–1.3)	1.1 (0.9–1.3)	1.0 (0.9–1.2)	1.0 (0.9–1.3)
Triglycerides, mmol/L	2.0 (1.5–2.7)	1.9 (1.2–2.9)	1.9 (1.4–2.9)	1.9 (1.4–2.8)	1.9 (1.4–2.9)	1.9 (1.4–2.9)
SGLT2is	25 (20.3)	26 (21.8)	102 (23.6)	83 (18.6)	150 (12.4)	164 (13.7)
CV medications						
Beta blockers	62 (50.4)	53 (44.5)	212 (49.1)	228 (51.1)	638 (52.7)	650 (54.1)
ACE inhibitors	55 (44.7)	50 (42.0)	171 (39.6)	159 (35.7)	398 (32.9)	406 (33.8)
ARBs	66 (53.7)	67 (56.3)	251 (58.1)	274 (61.4)	749 (61.8)	719 (59.9)
Calcium channel blockers	58 (47.2)	71 (59.7)	229 (53.0)	243 (54.5)	733 (60.5)	697 (58.0)
ARNIs	1 (0.8)	4 (3.4)	1 (0.2)	4 (0.9)	9 (0.7)	5 (0.4)
Aldosterone antagonists	9 (7.3)	3 (2.5)	35 (8.1)	38 (8.5)	91 (7.5)	80 (6.7)
Lipid-lowering drugs						
Statins	94 (76.4)	85 (71.4)	323 (74.8)	331 (74.2)	933 (77.0)	923 (76.9)
Ezetimibe	4 (3.3)	5 (4.2)	32 (7.4)	33 (7.4)	83 (6.9)	94 (7.8)
PCSK9 inhibitors	0	2 (1.7)	0	1 (0.2)	2 (0.2)	4 (0.3)
Diuretics						
Thiazides	26 (21.1)	17 (14.3)	79 (18.3)	85 (19.1)	193 (15.9)	213 (17.7)
Loop diuretics	15 (12.2)	15 (12.6)	73 (16.9)	84 (18.8)	340 (28.1)	363 (30.2)
Thiazide-like diuretics	15 (12.2)	17 (14.3)	56 (13.0)	54 (12.1)	104 (8.6)	117 (9.7)
Acetylsalicylic acid	57 (46.3)	51 (42.9)	194 (44.9)	216 (48.4)	500 (41.3)	509 (42.4)
P2Y12 inhibitors	12 (9.8)	11 (9.2)	48 (11.1)	42 (9.4)	136 (11.2)	144 (12.0)
Other	3 (2.4)	3 (2.5)	11 (2.5)	12 (2.7)	22 (1.8)	23 (1.9)
DOAC	7 (5.7)	12 (10.1)	28 (6.5)	30 (6.7)	76 (6.3)	75 (6.2)
Vitamin K antagonists	2 (1.6)	2 (1.7)	22 (5.1)	16 (3.6)	30 (2.5)	36 (3.0)
Atrial fibrillation	5 (4.1)	12 (10.1)	42 (9.7)	33 (7.4)	87 (7.2)	88 (7.3)
Heart failure						
HFpEF	10 (8.1)	15 (12.6)	39 (9.0)	37 (8.3)	118 (9.7)	106 (8.8)
HFrEF	4 (3.3)	4 (3.4)	19 (4.4)	11 (2.5)	39 (3.2)	46 (3.8)
No heart failure	97 (78.9)	93 (78.2)	352 (81.5)	373 (83.6)	974 (80.4)	964 (80.3)
Unknown	12 (9.8)	7 (5.9)	22 (5.1)	25 (5.6)	79 (6.5)	85 (7.1)
LVEF						
<40%	1 (0.8)	3 (2.5)	12 (2.8)	8 (1.8)	26 (2.1)	33 (2.7)
40 to <50%	4 (3.3)	4 (3.4)	13 (3.0)	9 (2.0)	29 (2.4)	45 (3.7)
≥50%	33 (26.8)	32 (26.9)	139 (32.2)	138 (30.9)	360 (29.7)	363 (30.2)

Values are *n* (%) or median (interquartile range). One subject in the semaglutide group had an unknown KDIGO group at baseline.

ACE, angiotensin-converting enzyme; ARB, angiotensin receptor blocker; ARNI, angiotensin receptor-neprilysin inhibitor; BMI, body mass index; CV, cardiovascular; DOAC, direct oral anticoagulant; eGFR, estimated glomerular filtration rate; HbA_1c_, glycated haemoglobin; HDL, high-density lipoprotein; HFpEF, heart failure with preserved ejection fraction; HFrEF, heart failure with reduced ejection fraction; hsCRP, high-sensitivity C-reactive protein; KDIGO, Kidney Disease Improving Global Outcomes; LDL, low-density lipoprotein; LVEF, left ventricular ejection fraction; MI, myocardial infarction; SGLT2i, sodium–glucose co-transporter 2 inhibitor; UACR, urine albumin-to-creatinine ratio.

**Figure 1:**
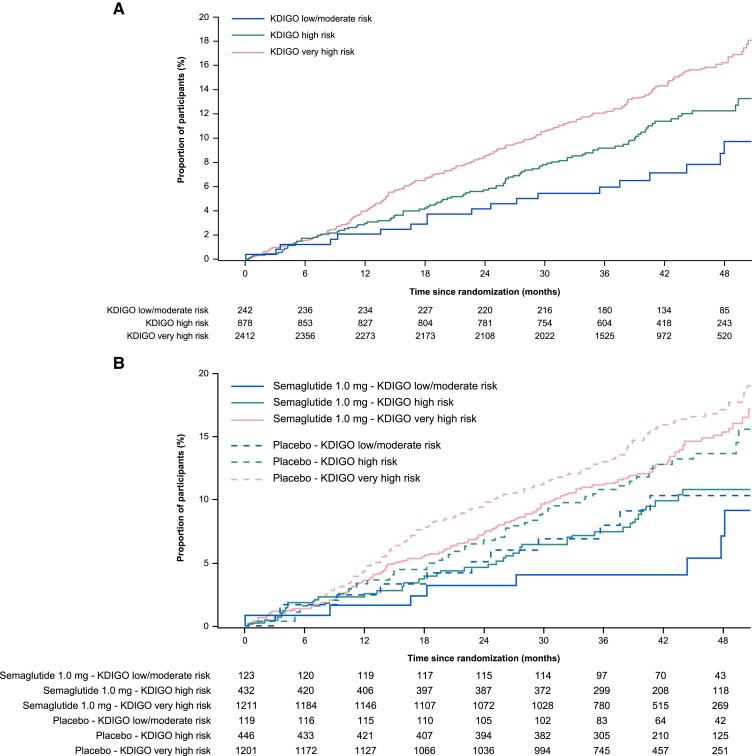
Time from randomization to first cardiovascular death/myocardial infarction/stroke event by (*A*) KDIGO risk class and (*B*) KDIGO risk class and randomized treatment. The cumulative incidence rate is calculated using Aalen–Johansen method with non-cardiovascular death as a competing risk. KDIGO, Kidney Disease Improving Global Outcomes

**Figure 2:**
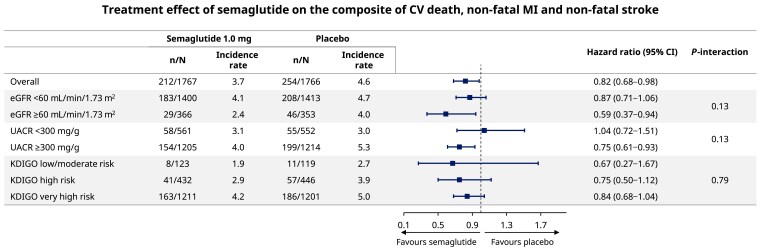
Effect of semaglutide 1.0 mg on the composite of CV death, non-fatal MI, and non-fatal stroke by baseline chronic kidney disease status. CI, confidence interval; CV, cardiovascular; eGFR, estimated glomerular filtration rate; KDIGO, Kidney Disease Improving Global Outcomes; MI, myocardial infarction; UACR, urine albumin-to-creatinine ratio

The time from randomization to first CV death/MI/stroke event by KDIGO risk class is presented in *Figure 1A*. *Figure 1B* shows the time to first CV/MI/stroke event by KDIGO risk class and randomized treatment assignment. Overall, those with a higher KDIGO risk class had higher rates of CV death/MI/stroke, with these events occurring in 19 participants (7.9%) with low/moderate risk, 98 (11.2%) with high risk, and 349 (14.5%) with very high risk over a median (range) follow-up of 3.4 years (0–4.5). *Figure 2* shows the effect of semaglutide compared with placebo on the composite of CV death/MI/stroke by baseline CKD status defined by eGFR, UACR, and KDIGO risk class. In the overall population, semaglutide reduced rates of the composite of CV death/MI/stroke compared with placebo [HR 0.82 (95% CI 0.68–0.98)]. Consistent treatment effects were observed across all CKD categories defined by eGFR, UACR, or KDIGO risk class (*P*-interaction >.05). Supplementary data online, *Figure S1*, shows CV death/MI/stroke composite and the individual components of the composite by more refined eGFR subgroups defined by eGFR <30, ≥30 to <45, ≥45 to <60, and ≥60 mL/min/1.73 m^2^. The number of participants was small in these subgroups, and the CIs were broad, but the *P*-interaction values for treatment by eGFR subgroup were non-significant.

**Figure 3:**
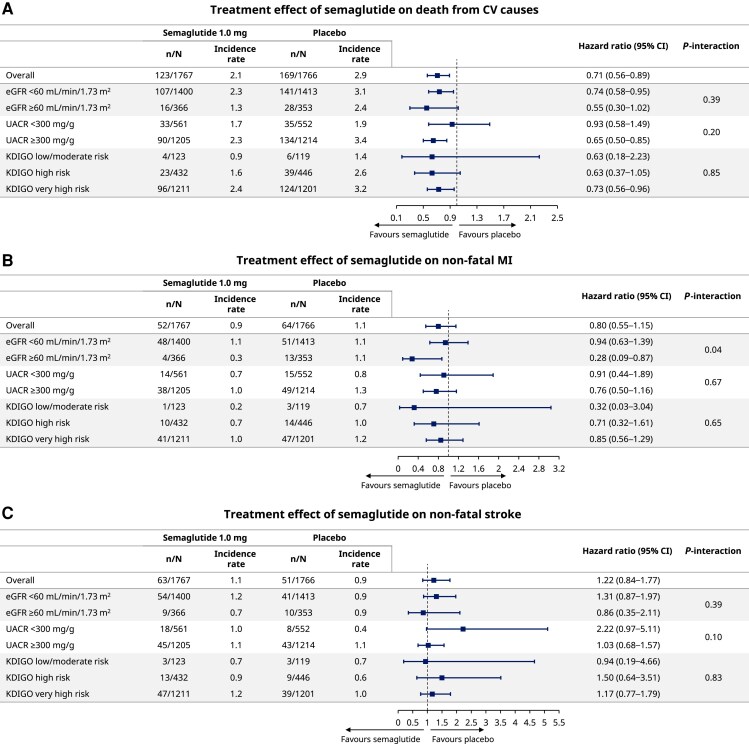
Effect of semaglutide 1.0 mg on (*A*) CV death, (*B*) non-fatal MI, and (*C*) non-fatal stroke by baseline chronic kidney disease. CI, confidence interval; CV, cardiovascular; eGFR, estimated glomerular filtration rate; KDIGO, Kidney Disease Improving Global Outcomes; MI, myocardial infarction; UACR, urine albumin-to-creatinine ratio

*Figure 3* shows the proportions of participants with events, incidence rates, and treatment effects for the individual components of the composite outcome of time to first CV death/MI/stroke. The number of MI and stroke events was lower than the number of CV deaths. Trends towards fewer MI events and more stroke events were observed for semaglutide compared with placebo. Overall, consistent treatment effects with semaglutide were observed for CV death/MI/stroke, with all *P*-interaction values >.05 except for the non-fatal MI and CKD defined by eGFR (*P*-interaction .04), for which the benefit observed appeared to be less in participants with eGFR <60 vs. ≥60 mL/min/1.73 m^2^ [HR 0.94 (95% CI 0.63–1.39) vs. HR 0.28 (95% CI 0.09–0.87), respectively].

**Figure 4:**
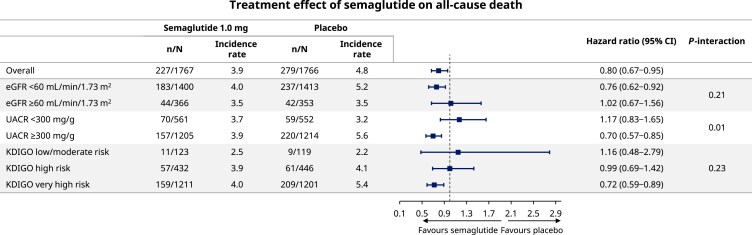
Effect of semaglutide 1.0 mg on death due to any cause by baseline chronic kidney disease. CI, confidence interval; eGFR, estimated glomerular filtration rate; KDIGO, Kidney Disease Improving Global Outcomes; UACR, urine albumin-to-creatinine ratio

The effect of semaglutide on all-cause mortality by baseline eGFR, UACR, and KDIGO risk class is presented in *Figure 4*. The incidence rates for death were higher in groups with more severe CKD, whether defined by eGFR, UACR, or KDIGO risk class. All-cause mortality was reduced by semaglutide overall [HR 0.80 (95% CI 0.67–0.95)], with consistent results across CKD categories defined by eGFR (*P*-interaction .21) and KDIGO risk class (*P*-interaction .23). For UACR category, the randomized treatment effect on all-cause mortality favoured semaglutide in participants with higher UACR [HR 0.70 (95% CI 0.57–0.85) for UACR ≥300 mg/g] compared with a trend towards higher mortality with semaglutide in participants with lower UACR [HR 1.17 (95% CI 0.83–1.65) for UACR <300 mg/g] (*P*-interaction .01).

**Figure 5:**
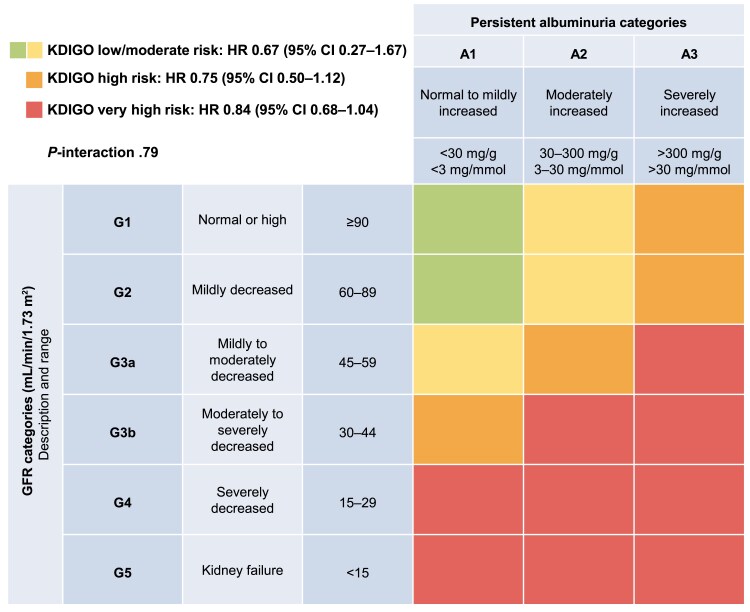
KDIGO heat map with composite cardiovascular death, non-fatal myocardial infarction, and non-fatal stroke event rates (semaglutide 1.0 mg vs. placebo). CI, confidence interval; G, grade; GFR, glomerular filtration rate; HR, hazard ratio; KDIGO, Kidney Disease Improving Global Outcomes

*Figure 5* shows the treatment effects of semaglutide by KDIGO risk class represented as a heat map. Similar reductions with semaglutide across KDIGO risk class were observed for the composite of CV death/MI/stroke, with HRs ranging from 0.67 to 0.84 and *P*-value for the interaction not significant (.79).

*Table 2* shows the key safety outcomes by KDIGO risk class for semaglutide and placebo. Participants with higher KDIGO risk class had a higher number of SAEs and fatal events. Although differences were marginal, the proportions of participants with SAEs were numerically lower for the semaglutide than the placebo group within each KDIGO risk category.

**Table 2:** Key safety outcomes by KDIGO risk class

	KDIGO low/moderate risk		KDIGO high risk		KDIGO very high risk	
	Semaglutide 1.0 mg (*n* = 123)	Placebo (*n* = 119)	Semaglutide 1.0 mg (*n* = 432)	Placebo (*n* = 446)	Semaglutide 1.0 mg (*n* = 1211)	Placebo (*n* = 1201)
SAEs	47 (38.2)	52 (43.7)	185 (42.8)	210 (47.1)	573 (47.3)	631 (52.5)
AEs leading to permanent discontinuation	14 (11.4)	12 (10.1)	51 (11.8)	45 (10.1)	167 (13.8)	152 (12.7)
Fatal events	9 (7.3)	7 (5.9)	43 (10.0)	51 (11.4)	121 (10.0)	155 (12.9)
Gallbladder disease	2 (1.6)	6 (5.0)	9 (2.1)	9 (2.0)	25 (2.1)	32 (2.7)
Pancreatitis	1 (0.8)	2 (1.7)	4 (0.9)	0	7 (0.6)	7 (0.6)
Retinal disorders	18 (14.6)	19 (16.0)	98 (22.7)	86 (19.3)	279 (23.0)	283 (23.6)
Pancreatic cancer	0	2 (1.7)	2 (0.5)	0	3 (0.2)	4 (0.3)
Abuse or misuse of trial product	0	0	1 (0.2)	1 (0.2)	0	2 (0.2)
Acute kidney injury or failure	4 (3.3)	4 (3.4)	21 (4.9)	26 (5.8)	128 (10.6)	145 (12.1)
GI disorders	23 (18.7)	8 (6.7)	81 (18.8)	41 (9.2)	200 (16.5)	123 (10.2)
COVID-19	26 (21.1)	22 (18.5)	82 (19.0)	100 (22.4)	220 (18.2)	244 (20.3)
Allergic reactions	3 (2.4)	1 (0.8)	7 (1.6)	8 (1.8)	23 (1.9)	29 (2.4)
CV disorders	13 (10.6)	10 (8.4)	59 (13.7)	69 (15.5)	186 (15.4)	227 (18.9)
Malignant tumours	14 (11.4)	6 (5.0)	25 (5.8)	21 (4.7)	64 (5.3)	62 (5.2)
Medication errors	1 (0.8)	0	6 (1.4)	3 (0.7)	11 (0.9)	8 (0.7)
Drug-related hepatic disorders	2 (1.6)	1 (0.8)	11 (2.5)	4 (0.9)	16 (1.3)	18 (1.5)

Values are *n* (%).

AE, adverse event; CV, cardiovascular; GI, gastrointestinal; KDIGO, Kidney Disease Improving Global Outcomes; SAE, serious adverse event.

The absolute risk reduction (semaglutide 1.0 mg − placebo) at Week 156 for CV death, MI, or stroke was −0.02 (95% CI −0.04 to −0.002; *P* = .035), resulting in an NNT of 45 (95% CI 23–623) to prevent one CV death, non-fatal MI, or non-fatal stroke. The absolute risk reduction at Week 156 for death due to any cause was −0.03 (95% CI −0.05 to −0.004; *P* = .019), resulting in an NNT of 39 (95% CI 21–238) to prevent one death.

## Discussion

In the FLOW trial of participants with T2D and CKD, subcutaneous semaglutide 1.0 mg once weekly slowed the decline in kidney function, improved kidney and CV outcomes, and reduced death from any cause compared with placebo.^21^ The present analyses support a clear and generally consistent benefit on CV outcomes and all-cause mortality across groups with varying severity of CKD at baseline defined by eGFR, UACR, and KDIGO risk class (*Structured Graphical Abstract*).

Participants with lower eGFR, higher UACR, or higher KDIGO risk class also had higher incidence of CV outcomes and all-cause mortality, a finding that is consistent with prior observations.^4,24^ The proportion of participants randomized to receive placebo who either died due to CV causes or had a non-fatal MI or non-fatal stroke over a median follow-up of only 3.4 years ranged from 9.2% in the KDIGO low/moderate risk class to 15.5% in the KDIGO very high risk class. The proportion of participants randomized to placebo who died due to any cause was also high and ranged from 7.6% in the KDIGO low/moderate risk class to 17.4% in the KDIGO very high risk class. Notably, compared with other dedicated kidney trials like CREDENCE,^9^ the FLOW trial population was at much higher risk, with an approximate 50% higher incidence of CV death/MI/stroke and death from any cause, although with slightly longer follow-up (3.4 vs. 2.6 years).

The FLOW trial is the first dedicated kidney outcomes trial in participants with T2D and CKD to evaluate a GLP-1RA. These analyses support the use of semaglutide to reduce CV outcomes and death due to any cause in T2D across a broad range of CKD severity. The results were observed in a population that was receiving a maximum labelled or tolerated dose of angiotensin-converting enzyme inhibitors or angiotensin receptor blockers, intensive lipid-lowering therapy (in >80% of participants), and antiplatelet or anticoagulant therapy. In addition, 15.6% of participants were receiving SGLT2i treatment; however, none were receiving finerenone at baseline because FLOW started enrolling in 2019 and finerenone trials were not completed until later. A prior systematic review has shown reductions in CV outcomes with GLP-1RAs in participants with eGFR ≥60 mL/min/1.73 m^2^ but not in those with eGFR <60 mL/min/1.73 m^2^.^25^ Similar to our findings, no heterogeneity in treatment effect with SGLT2i by CKD status defined by eGFR or albuminuria on CV death/MI/stroke was observed in a systematic review of completed SGLT2i trials and similarly with finerenone on CV death/MI/stroke/hospitalized HF.^26,27^ FLOW now clearly defines benefit in those with lower eGFR and supports use of semaglutide as part of disease management strategies for those with T2D and CKD. As treatment paradigms evolve, further study will be needed.

The analyses of the individual components of the CV composite outcomes showed that there were fewer non-fatal MIs and non-fatal strokes than CV deaths. This is consistent with prior dedicated kidney outcomes trials in T2D and CKD.^11,12^ A trend towards fewer MIs (52 vs. 64) but more strokes (63 vs. 51) was observed with semaglutide compared with placebo. Prior trials of semaglutide across different indications have not shown any potential increase in strokes,^28–30^ and systematic reviews of CV outcomes across GLP-1RA trials similarly have not shown a potential increase in strokes.^15–20,31^ In analyses of treatment by CKD severity with interactions for the composite CV outcome, the components of the composite outcome and all-cause mortality showed general consistency. Two of 15 interactions tested showed a significant *P*-interaction <.05. The *P*-interaction for the analysis of death due to any cause by UACR showed the randomized treatment effect on all-cause mortality favoured semaglutide in participants with higher UACR compared with a trend towards higher mortality with semaglutide in participants with lower UACR (*P*-interaction .01). In addition, the *P*-interaction for the analysis of non-fatal MI by eGFR showed an apparent larger treatment effect with semaglutide in participants with higher eGFR compared with those with lower eGFR (*P*-interaction .04). We are not aware of any biologically plausible mechanisms to explain these interactions, and given the number of interactions tested and the fact that we do not correct for multiple interactions, we believe this is likely due to chance.

These analyses have important limitations. The FLOW trial was not designed to have an adequate number of events or statistical power to definitively evaluate treatment effects in subgroups defined by CKD severity using eGFR, and the CIs are wide but no statistically significant interaction was observed. We did not analyse CKD defined by eGFR or UACR as continuous variables because of the small number of events in the extremes of kidney function that limited adequately powered analyses. The MI outcome in subgroups defined by eGFR and death due to any cause defined by UACR had *P*-interaction values <.05, suggesting greater efficacy in opposite directions by CKD severity measures. There is no clear biological reason for these findings, and given the large number of comparisons performed and opposite directions of effect, they are probably due to chance. Finally, these results are only applicable to patient populations reflective of the FLOW participants as defined by the inclusion CKD parameters applied.

In participants with T2D and CKD, semaglutide reduced the risk of CV death/MI/stroke and across subgroups defined by different levels of eGFR, albuminuria, or KDIGO risk class at baseline. Semaglutide reduced the risk of death from any cause consistently in subgroups defined by eGFR and KDIGO risk. Semaglutide should be considered as part of the therapeutic strategy to reduce CV risk in people living with T2D and CKD.

## Supplementary Material

ehae613_Supplementary_Data

## Declarations

### Data Availability

Data will be shared with bona fide researchers who submit a research proposal approved by the independent review board. Individual participant data will be shared in data sets in a deidentified and anonymized format. Information about data access request proposals can be found at novonordisk-trials.com.

### Pre-registered Clinical Trial Number

NCT03819153.
