# Supplementary material for: Cardiovascular outcomes with semaglutide by severity of chronic kidney disease in type 2 diabetes: the FLOW trial
Source: Eur Heart J. 2024 Aug 30;46(12):1096–108. doi: 10.1093/eurheartj/ehae613 (PMC11931213; doi:10.1093/eurheartj/ehae613)
Supplement: ehae613_Supplementary_Data [file ehae613_supplementary_data.docx]

**Supplementary material**

**Mahaffey KW, et al. Cardiovascular outcomes with semaglutide by severity of chronic kidney disease in type 2 diabetes: the FLOW trial**

**Supplementary Figure 1. Time from randomisation to first CV death, non-fatal MI, and non-fatal stroke by eGFR subgroup**


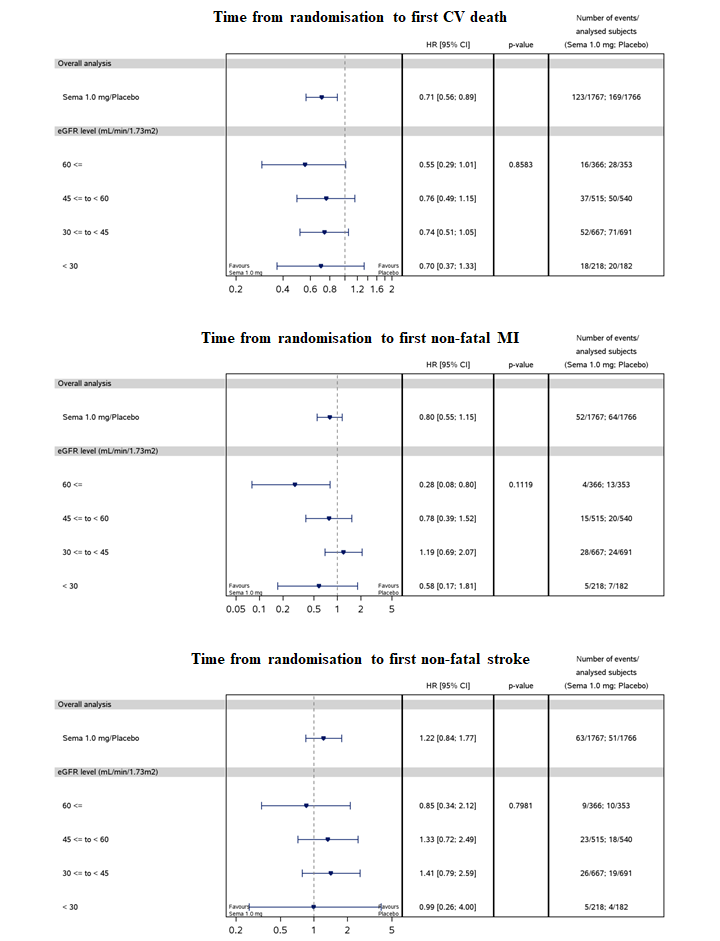


Data from the in-trial period. ASCVD comprised prior stroke or peripheral arterial disease.
Time from randomization to the relevant endpoint was analyzed using a Cox proportional hazards model with treatment as a fixed factor. Participants without events of interest were censored at the end of their in-trial period. For subgroup analyses, estimated HR and corresponding CIs were calculated using a Cox proportional hazards model with interaction between treatment group and subgroup as a fixed factor. The model was stratified by SGLT2i use at baseline.
p-value: p-value for the test of no interaction effect.
ASCVD, atherosclerotic cardiovascular disease; CI, confidence interval; eGFR, estimated glomerular filtration rate; HF, heart failure, HFpEF, heart failure with preserved ejection fraction; HFrEF, heart failure with reduced ejection fraction; HR, hazard ratio; LVEF, left ventricular ejection fraction; NA, not available; Sema, semaglutide.
